# Counting Down the 2020 Goals for 9 Neglected Tropical Diseases: What Have We Learned From Quantitative Analysis and Transmission Modeling?

**DOI:** 10.1093/cid/ciy284

**Published:** 2018-06-01

**Authors:** T Déirdre Hollingsworth

**Affiliations:** Big Data Institute, Li Ka Shing Centre for Health Information and Discovery, Nuffideld Department of Medicine, University of Oxford, United Kingdom

**Keywords:** mathematical modeling, transmission dynamics, neglected tropical diseases, elimination as a public health problem

## Abstract

The control of neglected tropical diseases (NTDs) has received huge investment in recent years, leading to large reductions in morbidity. In 2012, the World Health Organization set ambitious targets for eliminating many of these diseases as a public health problem by 2020, an aspiration that was supported by donations of treatments, intervention materials, and funding committed by a broad partnership of stakeholders in the London Declaration on NTDs. Alongside these efforts, there has been an increasing role for quantitative analysis and modeling to support the achievement of these goals through evaluation of the likely impact of interventions, the factors that could undermine these achievements, and the role of new diagnostics and treatments in reducing transmission. In this special issue, we aim to summarize those insights in an accessible way. This article acts as an introduction to the special issue, outlining key concepts in NTDs and insights from modeling as we approach 2020.

## Teaser

Mathematical modeling has informed the development of control policies for neglected tropical diseases. Here we summarize the insights from a number of studies and the articles included in this issue.

## NEGLECTED TROPICAL DISEASES

Neglected tropical diseases (NTDs) are a diverse group of infections identified by the World Health Organization (WHO) as diseases that predominantly infect low-income populations in tropical countries, causing a large burden of morbidity and some mortality, and thus perpetuate the cycle of poverty [1]. In 2012, the WHO declared ambitious targets to reduce the burden of these diseases by eliminating them as a public health problem by 2020 [1]. In support of these aspirations, a diverse consortium of donors, pharmaceutical companies, government agencies, and others made large commitments of funding, donated treatments, and other activities for 10 of these diseases in the London Declaration on NTDs [2]. Large morbidity gains have been made over recent years [3], and there are active discussions on how to exploit the likely synergies between the goals for NTDs and universal health coverage (UHC), a sustainable development goal (SDG; target 3.8) [4], in particular how to extend these gains to the hardest-to-reach or conflict-affected communities [5]. Of these 10 diseases, Guinea worm is targeted for eradication; the remaining 9 infections are targeted for elimination as a public health problem in some settings. The adjustment of strategies to achieve control of the 9 infections, informed by mathematical modeling, is the focus of this special issue.

## QUANTITATIVE ANALYSIS AND TRANSMISSION MODELING IN PUBLIC-HEALTH POLICY

Infectious disease modeling has an increasing role in public-health policy, with resulting challenges and successes [6]. Appropriate analyses can provide thorough investigation and interpretation of data, as well as identify where the knowledge gaps are most acute. Models can also be used to rigorize our thinking on the processes of infection and transmission and test hypotheses about the likely dynamics and epidemiology.

Although there has been ongoing research into modeling of NTDs [7, 8], this research has sometimes been limited by the extent of biological knowledge and data on which to base these models. The availability of more extensive data, together with strong partnerships between researchers in different fields, including by the NTD Modelling Consortium [9], has led to marked improvements in these efforts. Researchers have made contributions not only in informing treatment strategies but also in informing diagnostic development and the applicability of new tools or treatments and in understanding the natural history of disease.

However, in comparison with other infectious diseases, we still have limited epidemiological data on NTDs; thus, although we have performed formal model comparisons [10–19], there remain large uncertainties in processes and parameters that could have an impact on the dynamics, as highlighted below. This means that we need to be cautious about overstating our results, even when the policy need is acute. This presents us with the challenge of correctly calculating and communicating the uncertainties in these complex systems while still giving an accessible message to end users.

This article acts as an introduction for a special issue that aims to increase the accessibility of the results so far by summarizing insights from NTD models and identifying key themes for the control of these diseases. It should be noted that in this special issue and in this article we have focused on epidemiological analyses and modeling and have not extended our focus to geospatial, spatial dynamic, or health economic modeling, all of which have an important part to play in developing policy for infectious diseases. We focus on the role of interventions to reach the 2020 goals for NTDs.

The authors of this issue are aiming to increase the repeatability of our science. The code for the models used in this special issue were previously published alongside more technical articles [10–19] as supplementary information or on our website (www.ntdmodelling.org). The release of raw code is not a complete answer to accessibility and reproducibility, but it is a step in the right direction [20].

The diseases are usually divided into 2 groups based on the methods used to control them, and we have summarized our results in this way. The first group includes those diseases that are mainly controlled by intensified disease management (IDM) or increased detection, screening, and treatment of infection. The second group includes those diseases that are mainly controlled by mass drug administration (MDA). Although there are major epidemiological differences between diseases in these groups, they share some common uncertainties in informing control, which are discussed in each section.

## IMPROVED CASE FINDING AND TREATMENT

Leprosy, the Gambian form of sleeping sickness (human African trypanosomiasis), visceral leishmaniasis in the Indian subcontinent, and Chagas disease are four London Declaration NTDs which are characterized as IDM infections. They have long, variable periods between infection and symptomatic disease and, generally, late diagnosis. Control strategies are focused on reducing time to diagnosis and case finding with accompanying vector control, where appropriate.

**Figure 1.**
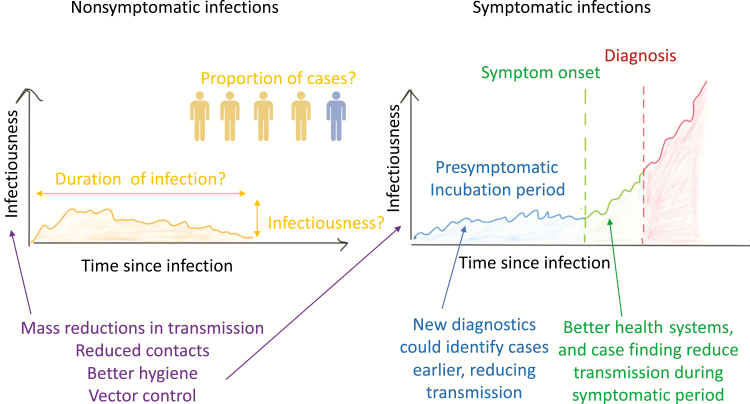
Schematic highlighting how uncertainties in the natural history of infections impact our estimates of transmission, and the role of different interventions in controlling them. Left: nonsymptomatic cases; right: symptomatic cases. We hypothesize a likely profile of infectiousness over time for each type of infectious individual. The area under this curve is proportional to the expected number of onward transmissions due to different stages of infection in a wholly susceptible population. If there are many nonsymptomatic people for each symptomatic individual, they may collectively contribute substantially to transmission even if their individual contribution is low (multiple yellow areas for single blue, green areas). For the symptomatic individuals, the relative infectiousness and duration of the symptomatic phase will determine the population-level impact of diagnosing and treating cases earlier (covering more of the green or even blue area). Vector control or other mass interventions could reduce both symptomatic and asymptomatic transmission by reducing all transmissions.

These diseases are characterized by long, uncertain incubation periods and an unknown degree of transmission by asymptomatic individuals (Figure 1). The potential role of asymptomatic individuals in transmission is well known in epidemiology. The close link between symptoms and infectiousness for smallpox and severe acute respiratory syndrome (SARS) has been calculated to be crucial in controlling these diseases [21]. That analysis explicitly considers the relative contribution of asymptomatics in terms of their contribution to the number of onward transmissions that an individual would be responsible for during a new outbreak, or the basic reproduction number, *R*_*0*_, in a way that is implicitly included in many models, but less elegantly presented.

We could adopt a similar framework for these NTDs. First we would need to separate asymptomatics into presymptomatic and nonsymptomatic (Figure 1, yellow and blue), highlighting the problems in language around asymptomatics [22]. For nonsymptomatic individuals yellow, left hand plot (Figure 1), infectiousness may rise and fall but is generally expected to be low, based on pathogen measurements. A key question is, of course, the relationship between these pathogen measurements and transmission, which is unknown and likely to be nonlinear.

The contribution of a single nonsymptomatic individual to transmission is proportional to the area under the infectivity since infection curve (yellow area, Figure 1), which might be large or small when compared with symptomatic infection. A similar calculation has contributed to the debate in human immunodeficiency virus (HIV) around the relative roles of the early, brief period of high infectivity when compared with the much longer period of asymptomatic infection with lower transmission rates [23–25]. Unfortunately, every aspect of infectivity and duration of infection (Figure 1) is highly uncertain for these 4 NTDs.

Of course, it is not only the contribution of each nonsymptomatic individual to transmission that is important, but also the proportion of the infected population who fall into this group (Figure 1, yellow vs blue). It is also crucial to note that this contribution will change through the course of an epidemic and an intervention and will be dependent on the type of intervention being applied, as nicely illustrated for visceral leishmaniasis in this issue [26, 27]. If the symptomatic phase is highly infectious and of sufficient duration (Figure 1, right-hand plot, green and red), interventions to identify cases early in the symptomatic period are likely to be highly effective. Although Chagas may not follow the increasing infectivity over time pattern but instead have rather high infectivity during early acute infection, diagnosis is so rare that postsymptomatic infections are often treated as asymptomatic infections, which may contribute substantially to transmission [28].

In summary, the balance between infectivity, duration of infection, and frequency of asymptomatic versus symptomatic infection may undermine any attempt to control a disease solely by increased case finding. This can be mitigated by reducing all infectivity through vector control or other transmission-reducing interventions, which reduces the infectivity of all infected invidiuals (the height of the curve in Figure 1). Although this theoretical framework is useful, there is much modeling work to be done to populate a more concrete discussion of the relative roles of different phases of infections for these complex diseases. The details of each infection are, of course, very important and should be considered individually.

Leprosy, a directly transmitted bacterial infection, was one of the first NTDs to have global targets for elimination as a public health problem, leading to large declines, although these have stabilized in the last decade [29–31]. Leprosy exemplifies the problem with surveillance for a disease in which cases are identified if both the infected individuals seek care and the appropriate care is available for them and mathematical modeling provides methods for estimating the pool of undiagnosed infections [32]. In addition, modeling has highlighted the need for much earlier diagnosis and suggests that targeted case finding through household contact tracing, perhaps combined with postexposure prophylaxis, could hold great potential for control (Table 1).

**Table 1.** Summary of Recommendations From Modeling for 4 Neglected Tropical Diseases Controlled Primarily by Intensified Disease Management

Disease	Current strategy	Key elimination strategies	Programmatic considerations
Gambiense sleeping sickness [34]	Active screening using mobile teams Passive detection in fixed health facilities	• Tsetse control using tiny targets to accelerate breaking transmission • Enhancing passive detection by increasing access to HAT diagnostics • Targeting high-risk groups and increasing turn-out in active screening	• Large-scale deployment and maintenance of targets in hard-to-reach regions • How to identify and target high-risk groups to screen
Visceral leishmaniasis in the Indian subcontinent [27]	4 phases of interventions: preparatory phase, 5-year attack phase with ACD and high-coverage IRS, ≥ 3-year consolidation phase with limited IRS and intensified ACD, maintenance phase to ensure elimination target sustained	• Adjust attack phase duration according to precontrol endemicity (eg, increase duration for high precontrol endemicity settings) • Carry out active case detection and treatment of PKDL cases • Include PKDL in elimination target	• As incidence decreases, the pool of susceptible individuals will grow, creating the potential for new large-scale outbreaks • Although potentially resource-saving, adjusting the attack phase duration by setting may be difficult to achieve in practice • Diagnosis of PKDL is challenging • An empirical threshold is required to include PKDL in the elimination target, and it is unclear what this should be
Chagas disease [36]	Vector control (indoor residual spraying) for domiciliated vectors	• Improve efficacy of vector control • Improve access to diagnosis and etiological treatment • Combine both strategies where feasible	• Efficacy and effectiveness of vector control is difficult to measure in practice • Sylvatic vectors will hardly be affected by indoor residual spraying of insecticides • Diagnosis and treatment of mother and child may help prevent congenital transmission, but in cases of chronic Chagas disease, treatment may be perceived as noncurative and hence not adhered to (although parasite clearance effected by treatment would reduce onward transmission and have a population impact)
Leprosy [32]	Passive case detection in local health- care facilities Active case detection in mobile facilities (eg, “skin camps”) Active case detection in high prevalence communities and households	• Earlier case detection (eg, better diagnostics, more active surveillance) • Targeted active surveillance and prophylatic chemotherapy (eg, identification of transmission “hot spots”)	• Stigma remains a substantial barrier to early diagnosis • Migration and movement (the long period between infection and disease makes movement important) • Diagnostic procedures and prophylactic chemotherapy both need development

Abbreviations: ACD, active case detection; HAT, human African trypanosomiasis; IRS, indoor residual spraying of insecticide; PKDL, post–kala azar dermal leishmaniasis.

In constrast with the global scope of leprosy, the Gambian form of sleeping sickness (human African trypanosomiasis), which is transmitted by tsetse flies, is focused in Western Africa. It has a high case fatality rate and is targeted for elimination because it is thought to be an anthroponotic disease and current interventions have led to large drops in case numbers [33]. The main method of control is through screening of populations and treatment of infected individuals. A key question for sleeping sickness is the potential contribution of vector control as a complement to screening and treating. Modeling suggests that it could have a large impact, reducing transmission not only from cases but also from the uncertain quantity of asymptomatic or presymptomatic individuals, or even animal hosts [34]. The modelers also highlight the potential to increase the impact of screening, both passively, by increased access to diagnostics, and actively, by targeting high-risk groups (Table 1), to reduce the duration of infection and therefore transmission (the area under the curve in Figure 1) by all infected individuals.

Kala azar, or visceral leishmaniasis in the Indian subcontinent, is a parasite transmitted by sandflies, predominantly in the poorest communities. It poses a number of challenges for control, which consists of improving case detection and indoor residual spraying [35]. Cases are falling drastically, reducing the burden of disease, but there is debate around the drivers of this decline, the size and nature of any asymptomatic pool, and the risk of resurgence [35]. Modeling acts as a tool to investigate some of the possible scenarios and evaluate different policy interventions in response to them [22, 27]. In particular, the recent modeling of the different stages of the control effort suggests that there should be some accounting for underlying transmission rates when selecting interventions and that post-kala-azar dermal leishmaniasis (PKDL), a late-stage potentially highly infectious state, could undermine control and should be studied more closely (Table 1).

Chagas disease is an anthropozoonosis caused by the protozoan *Trypanosoma cruzi*, which is often contracted in childhood, when symptoms are rarely diagnosed; instead it is more commonly diagnosed through sequelle such as heart disease in adulthood [28]. There are huge complexities in the zoonotic life cycle in different settings, and modeling can be used to evaluate how different vector-control interventions are likely to affect transmission [36]. The modeling summarized in this issue highlights the value of vector control in reducing the infectiousness of all infected individuals, as well as the value of increasing diagnosis rates (Table 1).

Across the IDMs, the models demonstrate how key uncertainties in life history have the potential to undermine the impact of current control long term but that intelligent intervention design may be able to overcome them.

## MASS DRUG ADMINISTRATION

**Figure 2.**
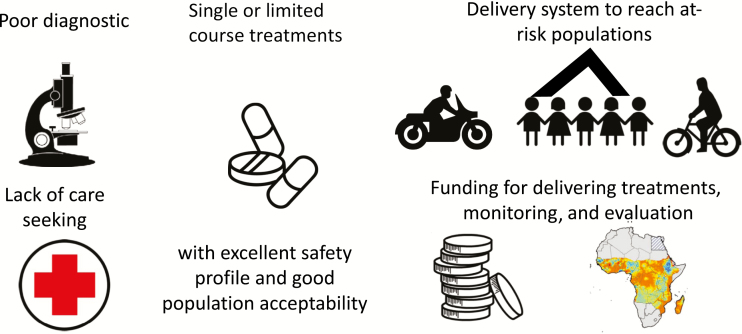
Schematic indicating the key building blocks that form the rationale for many mass drug administration campaigns for neglected tropical diseases.

A cornerstone of large-scale NTD control, specifically for lymphatic filariasis, onchocerciasis, soil-transmitted helminths, schistosomiasis, and trachoma, is MDA, sometimes in combination with vector control. An MDA program requires repeated distribution of treatments to large numbers of individuals, without diagnosis. They are therefore only considered when diagnosis of infection is difficult (eg, stool-based microscopy, night-time blood samples and microscopy), there is little care-seeking by infected individuals, and there is a treatment with an excellent safety profile with a straightforward or single treatment schedule (Figure 2). Donation of the treatments for these 5 diseases by the pharmaceutical manufacturers has transformed the opportunities for reducing the burden of these diseases, but it has required additional investment to deliver the treatments, as well as data to determine when and where MDA should be delivered.

**Figure 3.**
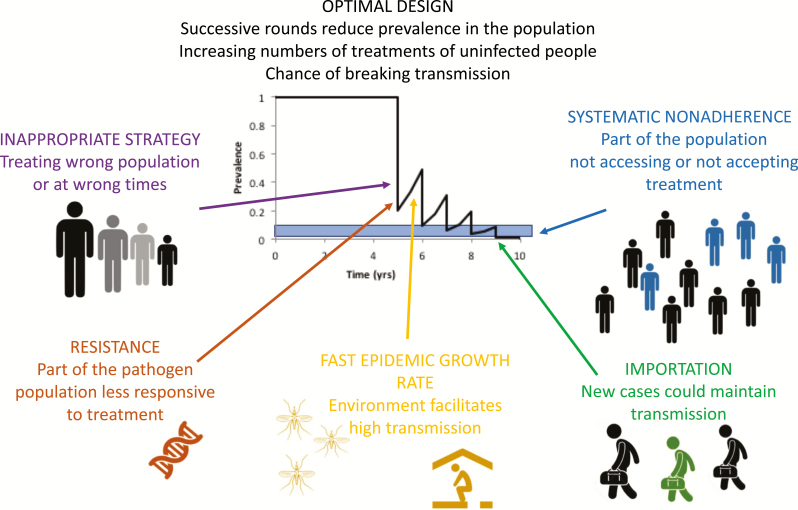
Schematic of factors that could undermine the success of a mass drug administration program. Monitoring and evaluation of programs is usually focused around a survey just prior to a round of treatment. If infection is not falling as quickly as expected, it could be due to any of the reasons outlined in the schematic, most of which cannot be detected by routine surveillance.

Through the course of a successful MDA, increasing numbers of treatments go to those uninfected at the time (Figure 3). Of course, these individuals are uninfected because they have been protected due to the ongoing MDA program, which is a measure of the program’s success. The issue of infectious asymptomatic individuals, which is a major concern for the IDM diseases, is not such an issue as asymptomatic people are regularly treated as part of the MDA. Therefore, the key questions are who to treat (eg, which age group), how often to treat, and when treatments can be stopped [37].

A number of things can lead to the failure of an MDA, all of which have been investigated using mathematical modeling, including in this issue (Figure 3). One of the important issues in program design, and which can undermine a program’s success, is which parts of the population should be treated (Figure 3). If the wrong group is treated, you may see reductions in burden in this group, but not in the population at large. This is discussed in 2 of the papers in this special issue. Soil-transmitted helminths are transmitted through helminth eggs in feces contaminating the environment [38], and schistosomiasis is caused by intestinal worms that are passed in feces or urine and contaminate the water. In the case of schistosomiasis, the eggs then go on to infect snails, the parasite is amplified and rereleased into the water, and humans are infected through contact with that water [39]. For both of these infections, current guidelines suggest that treatments should be targeted at children, with different frequencies according to prevalence. The modeling studies in this special issue suggest that the current guidelines might be altered slightly to optimize their impact, through either targeting adults or changing the thresholds for switching strategies [40, 41] (Table 2).

**Table 2.** Summary of Recommendations From Modeling for 5 Neglected Tropical Diseases Controlled Primarily by Mass Drug Administration

Disease	Current strategy	Key elimination strategies	Programmatic considerations
Soil-transmitted helminthiasis [40]	MDA to school-aged children and high-risk subgroups	• Community-wide MDA in all but low-prevalence settings • No reduction in treatment frequency at the midline evaluation point due to the risk of recrudescence and failure to meet morbidity goal	• Cost of expanding the treated population • Restriction of drug donations to SAC • Unknown risk of drug resistance
Schistosomiasis [41]	MDA to school-aged children and high-risk subgroups.	• Increasing treatment coverage in school-aged children and expanding treatment coverage to include adults • Or increasing treatment frequency in moderate- to high-prevalence regions.	• Cost of expanding the treated population • Restriction of drug donations to use in school- aged children and availability of praziquantel • Difficulties assessing adherence to treatment • Defining the optimal strategy for tailoring the intervention to infection prevalence
Lymphatic filariasis [43]	MDA of all eligible persons	• Increasing coverage and reducing systematic nonadherence• Using the triple-drug to accelerate declines in appropriate areas	• Addressing systematic nonadherence• Availability of drug donations for triple-drug
Onchocerciasis [44]	Annual MDA with ivermectin of population aged 5 years	• Alternative MDA strategies (enhanced coverage, increased frequency), with or without complementary vector control, depending on history of MDA and local transmission conditions (or baseline endemicity)	• Vector control is laborious but could have benefits • Need for in-depth knowledge of vector breeding site ecology and hydrological conditions in rivers to be treated with larvicides for vector control • Cost and appropriate and timely implementation of higher frequency MDA programs in low-resources settings
Trachoma [45]	Annual MDA of all individuals	• In areas that have not reached control goals after a decade of treatment, intensive targeting of residual core group • Investigating coverage	• Identification of areas where current strategy is not working • Efficient assessment and treatment of residual core group

Abbreviations: MDA, mass drug administration; SAC, school-aged children.

The 2 other helminths considered here, lymphatic filariasis, transmitted by mosquitoes and a risk factor for elephantiasis, and onchocerciasis, transmitted by black flies and the cause of river blindness, are also targeted for elimination through MDA, sometimes accompanied by vector control [42]. The lymphatic filariasis campaign has been particularly successful, with billions of treatments given and recent scale-back of treatment in areas where the targets have been met. The policy discussion is around how best to accelerate achievement of the goals using alternative treatment strategies and, in particular, when and where these strategies might be most appropriate [43] and how they might be combined with vector control to slow down the epidemic growth rate between rounds of MDA [44]. If the bounce back rate is too fast, or the interval between treatments is too long, this can lead to all the gains from the previous round being lost (Figure 3). For onchocerciasis, the programs are at the point of adapting their strategies to reach beyond the large morbidity gains achieved so far. The modeling work discusses the alternative strategies and the potential for MDA combined with vector control to accelerate or achieve elimination (Table 2).

All of the articles on MDA policies highlight the importance of the epidemiological setting, the appropriate group being targeted, and systematic nonadherence, where particular groups either do not have access to or are refusing treatment, and note that these are often poorly measured (Figure 3) [40, 41, 43–45]. The issue of systematic nonadherence has been highlighted in modeling studies for many years but has recently become a point of focus again [11, 46, 47].

For trachoma, a bacterial infection that can cause blindness and is transmitted through an uncertain combination of vectors and direct contact, the modelers highlight an additional aspect of MDA, which is resistance to the drugs used for mass treatment (Table 2) [48]. This is because the MDA is a single dose of a broad-spectrum antibiotic, and so the concerns about rapid emergence of resistance have been present since the beginning, but current evidence suggests that the selection pressure from this single dose may not be as high as feared. They also highlight the risk of resurgence due to importation of cases, which is a particular concern because of the rapid epidemic growth rate of trachoma [45]. Both of these issues are relevant for the other MDA campaigns (Figure 3), but the longer time between generations and hence the slower epidemic growth rates for the helminths mean that both resistance and re-emergence are likely to be slower than for trachoma. However, as these campaigns have been running for decades in some cases, it is important to consider.

In addition to these issues, there are outstanding questions around when and where to halt MDA campaigns, which future modeling will inform. Treatment has already been halted in some areas for the lymphatic filariasis campaign. Issues of ongoing residual transmission, albeit with a likely slow growth rate, are being addressed and the decision to stop is being reevaluated [49].

## MODELING FOR NEW INTERVENTIONS AND TOOLS

In this article, we have discussed 2 main interventions for the control of NTDs: IDM and MDA. These definitions are part of a shifting landscape that is dependent on a changing epidemiology, demography, and on the availability of new tools. For example, with an appropriate treatment with a good safety profile in uninfected and nonsymptomatic persons, a disease could move from case detection to MDA or, when combined with the right diagnostic, to a screen and treat infection. Similarly, as prevalence falls for MDA diseases, if the right diagnostic becomes available, addressing these diseases could shift to a test-and-treat campaign or even case management. One of the roles of modeling is to evaluate the likely impact of new tools, treatments, and diagnostics; this is an active area of ongoing research that is not stressed in this issue.

## LIMITATIONS

Modeling of NTDs is constrained by particularly limited data, as these articles highlight through presentation of uncertainty in predictions, sensitivity analyses, or scenario-based investigation. In contrast with many other infections, the dynamics of these diseases are also characterized by slow timescales, which mean that many qualitative behaviors are robust to these unknowns. However, it should be noted that these analyses should be viewed as a current state of our knowledge, and data from ongoing research have the potential to reduce some of these key uncertainties.

## DISCUSSION

Across these diverse diseases, there are a number of common themes.

- Interventions should be tailored to the environment in which they are used, which requires more intensive data but should deliver greater gains.
- Reaching the right populations and ensuring uptake of screening, treatment, or MDA is an essential part of any campaign, and models can indicate at what level of coverage or systematic nonadherence these campaigns are more likely to fail.
- For a number of diseases, the relative contribution of sustained vector control transmission is an area of current evaluation. Vector control has the potential to speed the gains due to other interventions and maintain the gains once the biomedical interventions have taken place, but it may only be needed in certain areas.

Despite the large number of biological unknowns or uncertainties for NTDs, the slower dynamics allow us to develop our insights as data become more available.

In summary, the modeling analyses in this special issue demonstrate that 2020 goals for NTDs are likely to be met in a large number of areas. They also indicate what additional interventions are likely to be required in higher transmission areas or areas with particular epidemiological features. As such, this represents state-of-the-art modeling in this area and provides actionable information for policy development.

## Notes

***Acknowledgements.*** The author would like to thank the editors and staff of *Clinical Infectious Diseases* for all their contributions in developing and delivering this special issue on behalf of all the authors in it. Their support, patience, and clear guidance have been invaluable. The author also thanks members of the NTD Modelling Consortium for discussions regarding their contributions to the special issue and their contributions to Tables 1 and 2.

***Disclaimer.*** The views, opinions, assumptions, or any other information set out in this article are solely those of the author and should not be attributed to the funders or any person connected with the funders.

***Financial support.*** This work was supported by the Bill and Melinda Gates Foundation in partnership with the Task Force for Global Health through the NTD Modelling Consortium (grant no OPP1053230) and the Children’s Investment Fund Foundation (UK).

***Supplement sponsorship.*** This article appears as part of the supplement “Reaching the 2020 Goals for Nine Neglected Tropical Diseases,” sponsored by the NTD Modelling Consortium.

***Potential conflicts of interest.*** The author reports no conflicts of interest. The author has submitted the ICMJE Form for Disclosure of Potential Conflicts of Interest. Conflicts that the editors consider relevant to the content of the manuscript have been disclosed.
